# Generation of Transgene-Free Maize Male Sterile Lines Using the CRISPR/Cas9 System

**DOI:** 10.3389/fpls.2018.01180

**Published:** 2018-09-07

**Authors:** Rongrong Chen, Qilong Xu, Yan Liu, Jiaojiao Zhang, Dongtao Ren, Guoying Wang, Yunjun Liu

**Affiliations:** ^1^Institute of Crop Sciences, Chinese Academy of Agricultural Sciences, Beijing, China; ^2^State Key Laboratory of Plant Physiology and Biochemistry, College of Biological Sciences, China Agricultural University, Beijing, China

**Keywords:** maize, CRISPR/Cas9, genome editing, male sterility, transgene-free

## Abstract

Male sterility (MS) provides a useful breeding tool to harness hybrid vigor for hybrid seed production. It is necessary to generate new male sterile mutant lines for the development of hybrid seed production technology. The CRISPR/Cas9 technology is well suited for targeting genomes to generate male sterile mutants. In this study, we artificially synthesized *Streptococcus pyogenes Cas9* gene with biased codons of maize. A CRISPR/Cas9 vector targeting the *MS8* gene of maize was constructed and transformed into maize using an *Agrobacterium*-mediated method, and eight T_0_ independent transgenic lines were generated. Sequencing results showed that *MS8* genes in these T_0_ transgenic lines were not mutated. However, we detected mutations in the *MS8* gene in F_1_ and F_2_ progenies of the transgenic line H17. A potential off-target site sequence which had a single nucleotide that was different from the target was also mutated in the F_2_ progeny of the transgenic line H17. Mutation in the *MS8* gene and the male sterile phenotype could be stably inherited by the next generation in a Mendelian fashion. Transgene-free *ms8* male sterile plants were obtained by screening the F_2_ generation of male sterile plants, and the MS phenotype could be introduced into other elite inbred lines for hybrid production.

## Introduction

Male sterility (MS) provides a useful breeding tool to harness hybrid vigor for hybrid seed production, saving labor, and time. MS can be classified into genic male sterility (GMS) and cytoplasmic male sterility (CMS), based on the inheritance pattern. CMS is usually caused by the mutation of a mitochondrial gene that leads to the defective function of plant mitochondria. According to the molecular mechanism of MS, CMS is classified into three types: CMS-T, CMS-C, and CMS-S (Kim and Zhang, 2018). CMS has been widely used in hybrid seed production in three-line systems. However, the application of CMS is limited because of few restorer line resources, instability of the CMS phenotype, and disease susceptibility of CMS-based hybrids (Weider et al., 2009).

Genic male sterile plants are usually generated by mutations of some nuclear gene, and these plants can be used for hybrid seed production in two-line systems. GMS is a more attractive alternative for hybrid breeding than CMS. In 1921, the first maize GMS mutant was identified (Eyster, 1921). Currently, more than 40 maize *ms* mutants have been reported. *Male sterility 44* (*MS44*) gene was identified to encode a lipid transfer protein that is mainly expressed in the tapetum, and *ms44* is a dominant mutant of the gene (Fox et al., 2017). *Male sterility 45* (*MS45*) encodes a strictosidine synthase which plays an important role in the biosynthesis of terpenoid indole alkaloids, and it is required for the formation of the cell wall of male gametophytes (Cigan et al., 2001). *Male sterility 32* (*MS32*) encodes a basic helix-loop-helix (bHLH) transcription factor that regulates the division and differentiation of anther cells (Moon et al., 2013). *Male sterility 26* (*MS26*) encodes a cytochrome P450 monooxygenase, and it plays a role in fatty acid metabolism (Djukanovic et al., 2013). *Male sterility 7* (*MS7*) was cloned by map-based cloning, and it was identified that the gene encodes a plant homeodomain finger transcription factor. The mutation of the *MS7* gene led to abnormal programed cell death (PCD) of tapetal cells (Zhang et al., 2018). *Male Sterility 8* (*MS8*) encodes a putative ß-1,3-galactosyltransferase, and it mainly affects the meiotic stage of anther development (Wang et al., 2013). In the previous report, it was shown that the *ms8* mutant plants have fewer branches, that they do not exert anthers, and that they have no viable pollen. The anthers of the *ms8* mutant plants had more and shorter epidermal cells, while they also had less and larger tapetal cells (Wang et al., 2010). The *ms45* mutant has been successfully used in a hybrid production platform called the seed production technology (SPT) (Wu et al., 2015). A multi-control sterility system based on the maize *ms7* mutant has also been developed (Zhang et al., 2018).

Recently, the clustered regularly interspaced short palindromic repeats (CRISPR)/CRISPR-associated protein 9 (Cas9) system has been used as an efficient genome editing tool. The CRISPR/Cas9 technology is easy to use, and it almost replaces the transcription activator-like effector nuclease (TALEN) technology and the zinc finger nuclease (ZFN) technology. The CRISPR/Cas9 system has been applied for gene targeting in various species, including rice, wheat, cotton, *Arabidopsis*, tobacco, soybean, and barley (Li et al., 2013; Shan et al., 2013; Jacobs et al., 2015; Gao et al., 2017; Kapusi et al., 2017). Several studies have reported the use of CRISPR/Cas9 in gene targeting in maize (Liang et al., 2014; Svitashev et al., 2015; Feng et al., 2016; Zhu et al., 2016; Shi et al., 2017).

Maize male sterile mutants can also be generated by targeted mutation of candidate genes. Novel *ms26* male sterile lines were generated by targeted mutagenesis of *MS26* using the redesigned I-CreI homing endonuclease or the CRISPR/Cas9 (Djukanovic et al., 2013;Svitashev et al., 2015). Novel maize *ms45* mutants have also been produced by targeted mutagenesis of the *MS45* gene (Svitashev et al., 2015). Mutation of the *ZmTMS5* gene using the CRISPR/Cas9 technology has generated maize *tms5* male sterile mutants that are thermosensitive (Li et al., 2017). In this study, we edited the maize *MS8* gene using the CRISPR/Cas9 system, and we obtained novel transgene-free male sterile *ms8* lines of maize. These mutant lines are valuable resources for maize hybrid seed production.

## Materials and Methods

### Construction of the sgRNA-Cas9 Expression Vector

The codons of *Streptococcus pyogenes Cas9 (SpCas9)* gene were optimized using maize biased codons (Liu and Scott, 2009). The codon-optimized gene was attached with two nuclear localization sequences (NLSs), MSERKRREKL and MISESLRKAIGKR, at the N-terminal and C-terminal ends, respectively. The *Cas9* gene was synthesized and cloned into the pUC57 vector by Sangon Biotech (Shanghai, China) to generate the pUC57-Cas9 vector. Using pUC57-Cas9 plasmid as the template, the *Cas9* fragment was amplified by PCR with the *Bsm*BI and *Nco*I sites added to the N-terminal and with the *Bst*EII site added to the C-terminal. The amplified *Cas9* fragment was digested with *Bsm*BI and *Bst*EII, and it was inserted into the pCAMBIA3301 plasmid at the *Nco*I and *Bst*EII sites to generate the pCAMBIA3301-Cas9 vector.

Maize U3 promoter sequence was also synthesized at Sangon Biotech (Shanghai, China), and it was ligated into the pUC57 vector to generate the pUC57-ZmU3 vector. The sequences of *Cas9* and the single guide RNA (sgRNA) construct were shown in **Supplementary File S1**.

**FIGURE 1:**
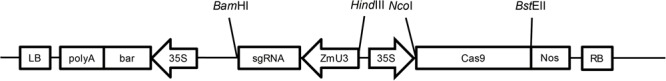
The T-DNA region of the pCAMBIA3301-Cas9-MS8ex2 plasmid. The codon-optimized *Streptococcus pyogenes Cas9* (*SpCas9*) gene was inserted into the pCAMBIA3301 plasmid at *Nco*I and *Bst*EII sites after the 35S promoter. The ZmU3::sgRNA was inserted into the *Bam*HI and *Hind*III sites. LB, T-DNA left border; polyA, 35S polyA terminator; bar, bar gene; 35S, CaMV 35S promoter; sgRNA, single guide RNA; ZmU3, *Zea mays* U3 promoter; Cas9, *Streptococcus pyogenes Cas9* gene; Nos, Nos terminator; RB, T-DNA right border; *Bam*HI, *Hin*dIII, *Nco*I, *Bst*EII are restriction enzyme sites.

A sgRNA targeting the second exon of *MS8* (Zm00001d012234) was designed as 5′-GCTGTCCGGGAAGGCCGTCGCGG-3′. Primers 5′-AGCAGCTGTCCGGGAAGGCCGTCG-3′ and 5′-AAACCGACGGCCTTCCCGGACAGC-3′ were synthesized at Sangon Biotech (Shanghai, China). The two primers were mixed and heated at 95°C for 5 min, annealed at room temperature, and then cloned into the pUC57-ZmU3 vector which was digested with *Bsa*I to construct the pUC57-ZmU3-MS8ex2 vector. The pUC57-ZmU3-MS8ex2 vector was digested with *Bam*HI and *Hin*dIII, and the ZmU3-MS8ex2 fragment was purified and inserted into the pCAMBIA3301-Cas9 plasmid to construct the pCAMBIA3301-Cas9-MS8ex2 transformation vector (**Figure 1**).

### *Agrobacterium*-Mediated Maize Transformation

The pCAMBIA3301-Cas9-MS8ex2 vector was transformed into the *Agrobacterium tumefaciens* strain EHA105. Immature embryos from maize hybrid HiII were transformed according to the described method (Zhang et al., 2013) with some modifications. Immature embryos about the size of 1.2 mm were isolated and suspended in liquid infection medium (Murashige and Skoog basal medium, 68.5 g L^-1^ sucrose, 36.0 g L^-1^ glucose, 100 μM acetosyringone, pH 5.2). The embryos were heated at 45°C for 3 min, and then transferred to an *A. tumefaciens* suspension and incubated for 5 min. After inoculation, the embryos were transferred to solid cocultivation medium (half strength Murashige and Skoog basal medium, 20 g L^-1^ sucrose, 10 g L^-1^ glucose, 0.85 mg L^-1^ silver nitrate, 100 μM acetosyringone, 1.22 mg L^-1^ CuSO_4_, 8 g L^-1^ agar, pH 5.8) and were incubated in the dark at 23°C for 3 days. The embryos were then transferred onto resting medium (Murashige and Skoog basal medium, 20 g L^-1^ sucrose, 10 g L^-1^ glucose, 1.5 mg L^-1^ 2,4-Dichlorophenoxyacetic acid (2,4-D), 0.85 mg L^-1^ silver nitrate, 250 mg L^-1^ cefotaxime, 8 g L^-1^ agar, pH 5.8) and were cultured at 28°C for 7 days. The embryos were moved to a selection medium (Murashige and Skoog basal medium, 20 g L^-1^ sucrose, 10 g L^-1^ glucose, 0.7 mg L^-1^ proline, 0.25 mg L^-1^ myo-inositol, 1.5 mg L^-1^ 2,4-D, 0.5 mg L^-1^ 6-Benzylaminopurine (6-BA), 1.22 mg L^-1^ CuSO_4_, 250 mg L^-1^ cefotaxime, 1.5 mg L^-1^ bialaphos, 8 g L^-1^ agar, pH 5.8) and were maintained for 2 weeks under dim light (10 μmol m^-2^ s^-1^) at 28°C. Then, bialaphos was increased to 3 mg L^-1^ in the selection medium for two rounds of 2-week selection. Resistant calli were placed in Murashige and Skoog medium containing 1 mg L^-1^ bialaphos for regeneration under fluorescent white light in a 16/8 h light/dark cycle. The regenerated shoots were transferred to Murashige and Skoog rooting medium. Two weeks later, the regenerated T_0_ seedlings were transferred to soil and were grown in a greenhouse with 16/8 h light/dark cycle at 25–28°C.

### Maize Propagation

F_1_ generation plants were produced by crossing the transgenic line H17 with the maize inbred line Zong31. These F_1_ plants were self-pollinated to produce the F_2_ generation. Backcrossing of the MS trait to maize inbred lines was performed by crossing transgene-free male sterile mutant plants *ms8*-6 or *ms8-*18 with maize inbred line Zheng58, respectively. The generated F_1_ plants were then self-pollinated to produce the F_2_ plants. The F_1_ and F_2_ plants were grown in the field in Beijing or Hainan Province, China.

### PCR Analysis of the Transgenic Plants

Genomic DNA was extracted from the leaves of transgenic maize plants using the cetyltrimethylammonium bromide (CTAB) method (Murray and Thompson, 1980). For determining the mutations in the target gene, fragments flanking the target site were amplified by PCR using the genomic DNA as the template. For the transgenic male fertile F_1_ generation plants, PCR products were cloned into *pEASY*^®^-T1 Simple Cloning vectors (TransGen, Beijing, China) and five randomly selected individual clones were sequenced, or the PCR products were directly sequenced. For the transgenic male sterile F_2_ generation plants, PCR products were directly sequenced. For the determination of transgene-free male sterile plants, several fragments at the T-DNA region of the pCAMBIA3301-Cas9-MS8ex2 vector were amplified by PCR with the genomic DNA as the template. The PCR products were analyzed by agarose gel electrophoresis. Primers are listed in **Supplementary Table S1**.

### Phenotype Observation

At the flowering stage, the tassels of wild type (WT) and mutants were photographed in the field in Beijing, China. The spikelets were photographed in the laboratory, and the anthers were observed using an Olympus SZX7.

### Inheritance Analysis of CRISPR-Mediated *ms8* Genes

F_2_ plants were grown in the field in Beijing, China, and the phenotype of F_2_ plants were determined at the flowering stage. The numbers of male sterile plants and male fertile plants were recorded and analyzed using a *χ*^2^-test in Microsoft Excel.

## Results

### Knockout of *MS8* Gene in the Progeny of Transgenic Maize Plants

After maize transformation, eight independent T_0_ transgenic lines were generated. These transgenic lines were confirmed by PCR analysis of both *Cas9* gene and *bar* gene (data not shown). To identify whether the *MS8* genes in these transgenic lines were mutated, the targeted region of the *MS8* gene was amplified and sequenced directly. Unexpectedly, sequencing results showed that none of these T_0_ transgenic lines were mutated at the *MS8* gene. These T_0_ transgenic plants could not be self-pollinated to generate T_1_ generation plants in the greenhouse; hence, they were crossed with maize inbred line Zong31 to produce F_1_ generation, which was then self-pollinated to produce F_2_ progeny. To determine the mutations in the target site in F_1_ plants, fragments flanking the target site were amplified by PCR and were directly sequenced. The sequencing chromatograms of all the 12 F_1_ plants had chaotic peaks after the target site (**Supplementary Figure S1**), indicating that there were hemizygous mutations in the *MS8* gene at the target site in these plants. These PCR products were then cloned into *pEASY*^®^-T1 Simple Cloning vectors (TransGen, Beijing, China), and five randomly selected individual clones were sequenced. The sequencing results confirmed that two types of mutations occurred. One was named as *ms8-*DelG that had a guanine nucleotide deletion, and the other was named as *ms8*-InA that had an adenine nucleotide insertion (**Supplementary Figure S1**). Both of these two mutation types resulted in frame shift and a non-functional MS8 protein. The genotypes of these F_1_ plants were shown in **Supplementary Table S2**.

**FIGURE 2:**
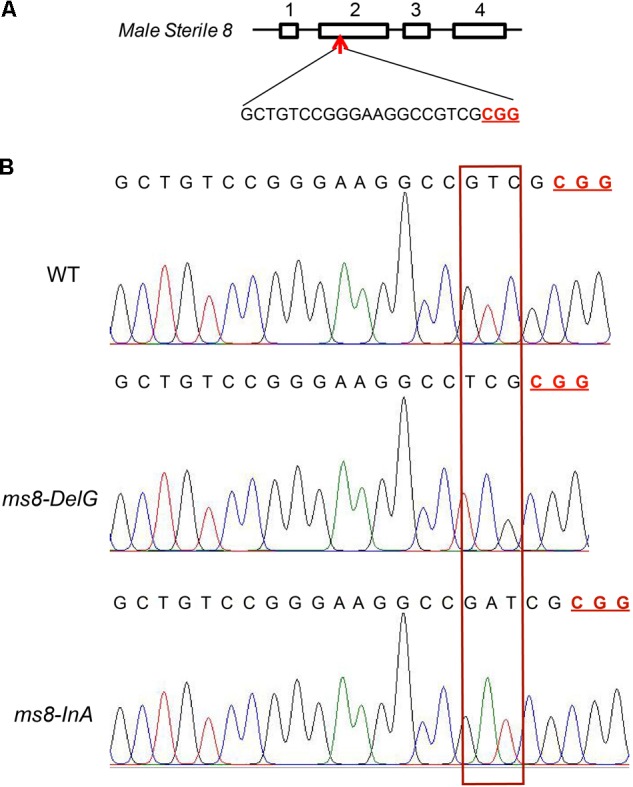
Gene-targeting of *MS8* gene in male sterile F_2_ plants. **(A)** Diagram of the *MS8* gene and the target site; four exons were numbered; the PAM motif is underlined and in bold. **(B)** The sequencing chromatograms of *MS8* in maize inbred line B73, *ms8*-DelG/*ms8*-DelG, and *ms8*-InA/*ms8*-InA plants. T_0_ transgenic line H17 plant was crossed with maize inbred line Zong31 to produce F_1_ generation, which was then self-pollinated to produce F_2_ progeny. Fragments flanking the target site were amplified by PCR using the genomic DNA of male sterile F_2_ plants as the template, and the PCR products were directly sequenced.

Among the 23 F_2_ progeny plants from the *ms8*-DelG-1 line and the 27 F_2_ progeny plants from the *ms8*-DelG-3 line (**Supplementary Table S2**), nine plants (*ms8*-1, *ms8*-2, *ms8*-3, *ms8*-4, *ms8*-5, *ms8*-6, *ms8*-7, *ms8*-8, and *ms8*-9) and six plants (*ms8*-19, *ms8*-20, *ms8*-21, *ms8*-22, *ms8*-23, and *ms8*-24) showed male sterile phenotype, respectively. Sequencing the *ms8* gene in these male sterile plants identified the homozygous mutation in *ms8-*DelG (**Figure 2**). Among the 27 F_2_ progeny plants obtained from line *ms8*-InA-2 (**Supplementary Table S2**), nine plants (*ms8*-10, *ms8*-11, *ms8*-12, *ms8*-13, *ms8*-14, *ms8*-15, *ms8*-16, *ms8*-17, and *ms8*-18) showed male sterile phenotype. Sequencing the *ms8* gene in these male sterile plants identified the homozygous mutation in *ms8-*InA (**Figure 2**). We grew more F_2_ progeny plants of these three lines to assess the ratio of male fertile and male sterile plants, and we found a 3:1 segregation between fertile and sterile plants (**Table 1**). This result suggested that the male sterile phenotype was inherited in a Mendelian fashion.

**Table 1:** Segregation analysis of the F_2_ generation produced by crossing transgenic T_0_ line H17 and inbred line Zong31.

Line	No. of male fertile plants	No. of male sterile plants	Expected ratio	*χ*^2^
*ms8*-DelG-1	104	39	3:1	0.28
*ms8*-InA-2	108	41	3:1	0.38
*ms8*-DelG-3	97	27	3:1	0.53

**FIGURE 3:**
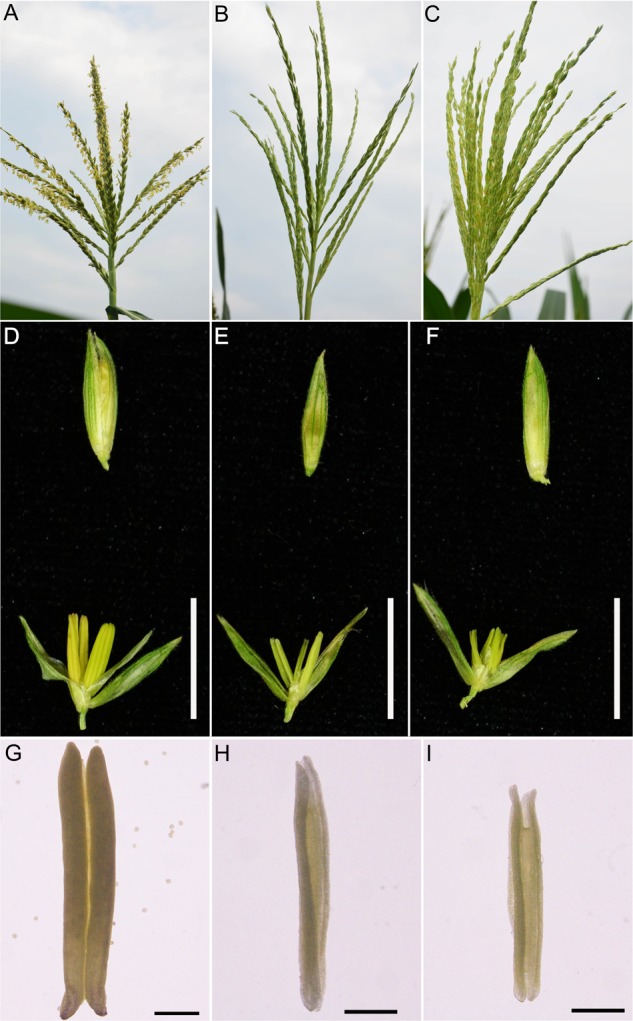
The phenotype of F_2_ mutant and wild type plants. The phenotype of tassels, spikelets, and anthers of WT **(A,D,G)**, *ms8-*DelG*/ms8-*DelG **(B,E,H)**, and *ms8*-InA/*ms8*-InA **(C,F,I)**. T_0_ transgenic line H17 plant was crossed with maize inbred line Zong31 to produce F_1_ generation, which was then self-pollinated to produce F_2_ progeny. At the flowering stage, the WT tassel had anthers, and the anthers were full of pollen, whereas tassels from the two mutants did not exert anthers, and anthers were empty without any pollen. Bar = 1 cm in **D–F**; bar = 1 mm in **G–I**.

Similar to WT plants, new *ms8* mutant plants have normal vegetative growth and female fertility. The phenotypes of these male sterile plants were significantly different from that of the WT at the flowering stage. The WT anthers were non-translucent because the anthers were full of pollen grains (**Figures 3A,D,G**), whereas none of the mutant plants had exerted anthers (**Figures 3B,C**). Mature mutant anthers were empty and half-translucent when viewed on a light table, without any visible pollen grains (**Figures 3E,F,H,I**). At the late stage, the mutant anthers began senescing and shrinking; meanwhile, the mutant tassel became white and eventually withered.

### Generation of Transgene-Free Male Sterile Plants

**FIGURE 4:**
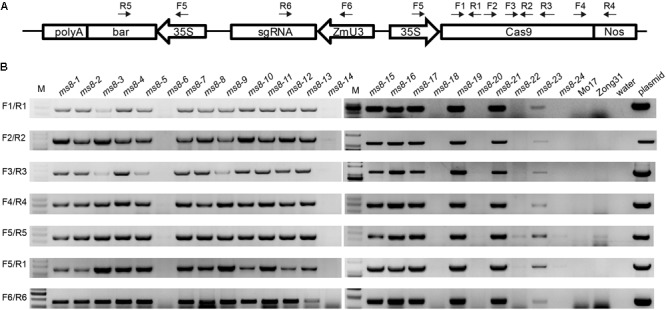
PCR screening of transgene-free male sterile F_2_ plants. **(A)** Schematic of the T-DNA region of the pCAMBIA3301-Cas9-MS8ex2 vector and the location of the primers. **(B)** Agarose gel electrophoresis results of seven fragments of T-DNA. M, DNA molecular weight marker; *ms8*-1–*ms8*-9: the male sterile F_2_ plants from the *ms8*-DelG-1 line; *ms8*-10–*ms8*-18: the male sterile F_2_ plants from the *ms8*-InA-2 line; *ms8*-19–*ms8*-24: the male sterile F_2_ plants from the *ms8*-DelG-3 line; Mo17: maize inbred line Mo17; Zong31: maize inbred line Zong31.

Because the MS phenotype was inherited in a Mendelian fashion, we speculated that transgene-free male sterile plants could be obtained in the progeny. To obtain transgene-free *ms8* male sterile plants, we used PCR to screen 15 F_2_ male sterile plants from the *ms8*-DelG-1 and *ms8*-DelG-3 lines. We also screened nine F_2_ male sterile plants from the *ms8*-InA-2 line. Six pairs of primers were designed to amplify *Cas9, sgRNA*, and *35S::bar* fragments (**Figure 4A**). Results showed that transgene fragments existed in 11 F_2_ mutant plants from the *ms8*-DelG-1 and *ms8*-DelG-3 lines, and seven F_2_ mutant plants from the *ms8*-InA-2 line, respectively. However, the transgene fragment was eliminated in four F_2_ male sterile plants from the *ms8*-DelG-1 and *ms8*-DelG-3 lines (*ms8*-6, *ms8*-20, *ms8*-22, and *ms8*-24) and in two F_2_ male sterile plants from the *ms8*-InA-2 line (*ms8*-14 and *ms8*-18) (**Figure 4B**). These results suggested that mutations of the *MS8* gene were fixed in these transgene-free plants.

To further investigate the inheritability of MS in these transgene-free mutants, we crossed two transgene-free male sterile plants *ms8*-6 and *ms8*-18, which belong to the *ms8*-DelG-1 and *ms8*-InA-2 lines, with maize elite inbred line Zheng58. All of the F_1_ progeny plants were male fertile, whereas the F_2_ population displayed a 3:1 segregation ratio between male fertile and male sterile plants (**Table 2**). These results suggested that the MS phenotype could be introduced to other elite inbred lines for hybrid production.

**Table 2:** Segregation analysis of the F_2_ generation produced by crossing transgene-free male sterile plants and inbred line Zheng58.

Line	Mutant plant	No. of male fertile plants	No. of male sterile plants	Expected ratio	*χ*^2^
*ms8*-DelG-1	*ms8*-6	73	16	3:1	1.98
*ms8*-InA-2	*ms8*-18	67	22	3:1	0.004

### Evaluation of Off-Target Mutations in Transgene-Free Male Sterile Mutants

Off-target mutations using the CRISPR/Cas9 system have been reported in many crops, including maize (Feng et al., 2016), rice (Endo et al., 2015), and soybean (Jacobs et al., 2015). To evaluate the possibility of off-target mutations induced by CRISPR/Cas9 in this study, we searched the potential off-target sites in the CRISPR-P website^1^. Nine potential off-target sites were found to have 95 or 85% identity with the target sequence. The fragments flanking four potential off-target sites were amplified from male sterile mutants and sequenced (**Table 3**). Sequencing results showed that mutations occurred in the potential off-target site sequence which had a single nucleotide that was different from the target. However, mutations did not occur in the sequences which had three nucleotides that were different from the target (**Table 3**). For the potential off-target site sequences which had a single nucleotide that was different from the target, a cytosine nucleotide deletion allele was identified in some male sterile plants, but the genotypes were not associated with the male sterile phenotype (**Supplementary Table S3**).

**Table 3:** The evaluation of off-target effects of CRISPR/Cas9.

Sequence of target site	Sequence of potential off-target sites	Off-target mutation	Loci of the potential off-target sites
GCTGTCCGGGAAGGCCGTCG**CGG**	GCTGTCGGGGAAGGCCGTCG**CGG**	Yes	Chr3:Zm00001d042639 CDS
	ATTGTCCGGGAACGCCGTCG**TGG**	No	Chr6:Intergenic
	GATGTCCAGGAAGCCCGTCG**CGG**	No	Chr4:Zm00001d051778 CDS
	GCTGGGCGAGAAGGCCGTCG**TGG**	No	Chr8:Zm00001d009511 CDS

*The PAM motif is underlined and the mismatched bases are in bold.*

## Discussion

Compared with CMS, GMS plant materials are much more useful for the hybrid SPT. Environment-sensitive GMS has been explored in two-line hybrid production system for wheat and rice (Whitford et al., 2013; Huang et al., 2014). Using GMS mutant *ms45*, DuPont Pioneer developed a hybrid production platform named SPT (Wu et al., 2015). Zhang et al. (2018) also developed a multi-control sterility system based on the *ms7* mutant. Expanding male sterile germplasm resources is necessary for developing new SPT technology. The MS phenotype is usually controlled by the recessive mutation of nuclear gene; therefore, it is possible to generate male-sterile mutants using the CRISPR/Cas9 system. Several MS genes have been identified to control the development of pollen or anther. In this study, we chose *male sterility 8* (*MS8*) as the targeted gene and generated novel *ms8* mutant lines using the CRISPR/Cas9 system. The MS phenotype of the novel *ms8* lines generated in this study could be introduced to other elite inbred lines for hybrid production, which is essential for the use of these mutants in producing hybrids. The new *ms8* lines generated in this study showed a male sterile phenotype similar to the reported natural *ms8* mutant in which the *Mu* transposon was inserted in the *MS8* gene (Wang et al., 2013).

If the CRISPR/Cas9 system works well, then there should be high editing efficiency in T_0_ generation plants. In this study, we did not observe mutations in the *MS8* gene in the T_0_ plants. T_0_ plants were crossed with maize inbred line Zong31 to generate F_1_ plants, and the editing of *MS8* gene in F_1_ plants was observed. The main reason for the low mutation efficiency of CRISPR/Cas9 system might be that the sgRNA is not stable and has poor targeting efficiency. It has been shown that the low activity of 35S promoter in germline cells would lead to low editing efficiency (Fauser et al., 2014). Using meiotic promoter to drive the expression of *Cas9* could increase the targeted mutagenesis efficiency (Eid et al., 2016; Mao et al., 2016). In this study, the *Cas9* gene was driven by the 35S promoter and its activity might be low. To localize the Cas9 protein into the nucleus, we fused the amino acids MSERKRREKL and MISESLRKAICKR (Shieh et al., 1993) to the N- and C-terminal ends of the Cas9 protein. We speculate that these nuclear location sequences (NLS) might not completely transport the target Cas9 protein to the nucleus, which might be another reason for the low mutation efficiency.

Off-target events often occurred during the gene targeting process using the CRISPR/Cas9 system. To reduce off-target editing, several strategies such as SpCas9-HF1 variants (Kleinstiver et al., 2016) and CRISPR/Cas9 nickase have been used (Shen et al., 2014). In our study, off-target mutations occurred in sequence which had a single nucleotide that was different from the target. However, there were no mutations in the sequences which had three nucleotides that were different from the target sequence. In some cases, off-target mutations might lead to the defective growth of plants. In this study, the off-target mutations in the *ms8* mutant did not lead to a negative effect on the mutant plants. Off-target mutations could also be eliminated by using the breeding methods of backcrossing or outcrossing.

Globally, there is a strict regulatory framework for transgenic crops. Using preassembled CRISPR/Cas9 ribonucleoproteins (RNPs) could generate transgene-free plants with edited genomes (Woo et al., 2015). CRISPR/Cas9 RNPs have also been successfully used to produce transgene-free genome-edited maize (Svitashev et al., 2016) and wheat (Liang et al., 2017). For the genome-edited crops, transgenes and off-targeted genes could also be eliminated by outcrossing or backcrossing. In general, the regulations for genome-edited crops without transgenes should be different from the traditional transgenic crops (Huang et al., 2016). In our study, transgenes were eliminated by backcrossing and transgene-free male sterile mutants were generated. These transgene-free male sterile mutants could be used for maize hybrid production systems without the limitation of regulatory frameworks for transgenic organisms. For application purposes, the MS phenotype of transgene-free mutants should be introduced into different elite maize lines by backcrossing.

## Conclusion

In this study, a CRISPR/Cas9 vector targeting the *MS8* gene of maize was constructed, and it was transformed into maize plants using an *Agrobacterium*-mediated method; consequently, we detected mutations in the *MS8* gene in F_1_ and F_2_ progeny plants. Mutations in the *MS8* gene and the male sterile phenotype could be stably inherited by the next generation in a Mendelian fashion. Transgene-free *ms8* male sterile plants were obtained, and the MS phenotype could be introduced into other elite inbred lines for hybrid production.

## Author Contributions

RC, GW, and YJL designed the research and wrote the article. RC, QX, YL, JZ, and DR performed the research and analyzed the data.

## Conflict of Interest Statement

The authors declare that the research was conducted in the absence of any commercial or financial relationships that could be construed as a potential conflict of interest.

## Footnotes

1 http://crispr.hzau.edu.cn/CRISPR2

## Supplementary Material

FIGURE S1 Gene-targeting of *MS8* gene in F_1_ plants produced by crossing T_0_ transgenic line H17 and inbred line Zong31. **(A)** The sequencing chromatograms of maize inbred line B73; **(B,C)** the sequencing chromatograms of *ms8-*DelG/*MS8* plant; **(D,E)** The sequencing chromatograms of *ms8-*InA/*MS8* plant. In **(B,D)**, PCR products were directly sequenced. In **(C,E)**, PCR products were cloned into *pEASY*^®^-T1 Simple Cloning vectors (TransGen, Beijing, China) and the randomly selected individual clone was sequenced. The PAM motif is underlined and in bold.

TABLE S1 Primers used in this study.

TABLE S2 The genotypes of F_1_ plants crossing between transgenic line H17 with inbred line Zong31.

TABLE S3 The genotypes of the off-target site with a single nucleotide difference.
