# Supplementary figures and images for: Generation of Transgene-Free Maize Male Sterile Lines Using the CRISPR/Cas9 System
Source: Front Plant Sci. 2018 Sep 7;9:1180. doi: 10.3389/fpls.2018.01180 (PMC6137208; doi:10.3389/fpls.2018.01180)

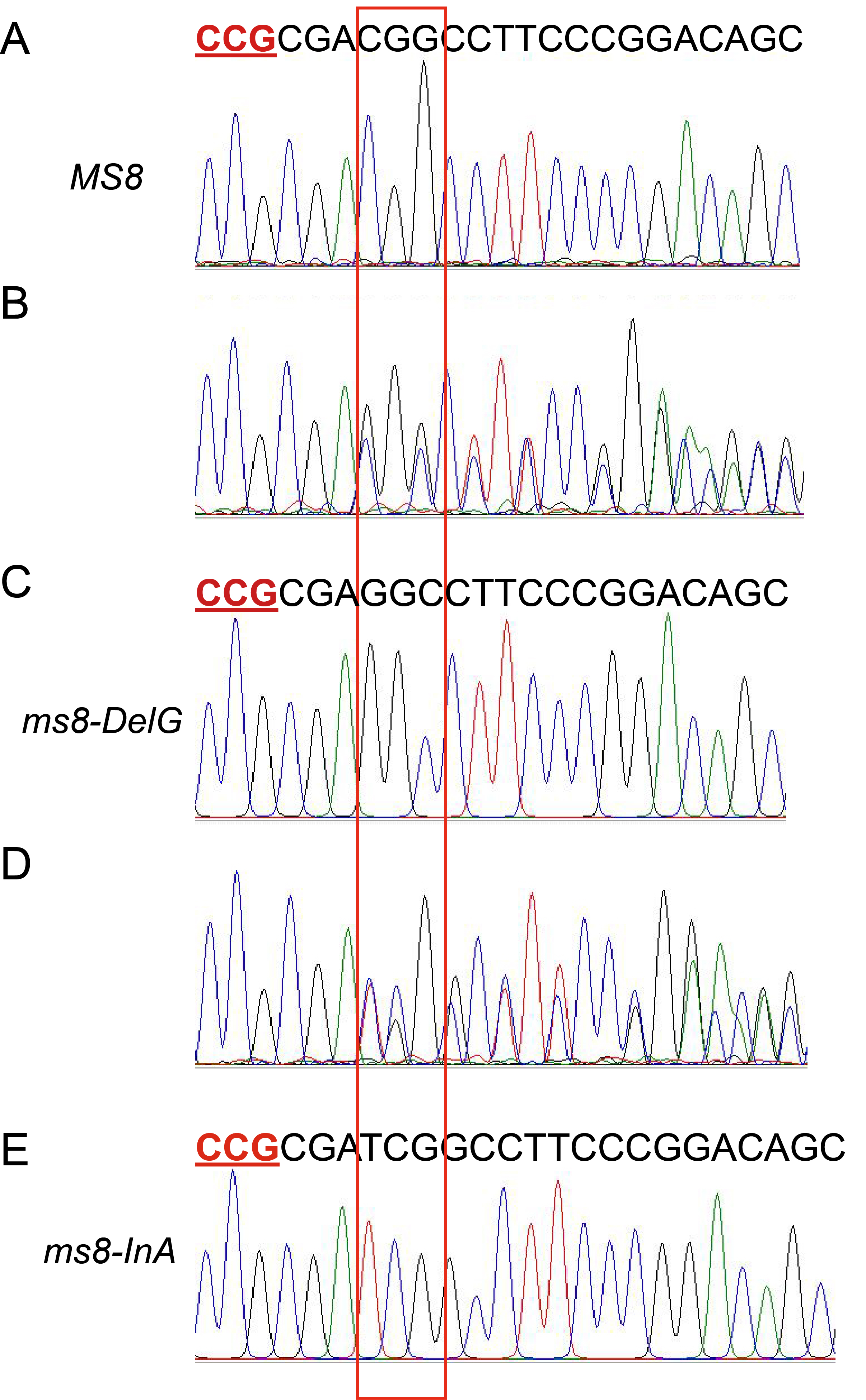

Supplement: FIGURE S1 — Gene-targeting of MS8 gene in F1 plants produced by crossing T0 transgenic line H17 and inbred line Zong31. (A) The sequencing chromatograms of maize inbred line B73; (B,C) the sequencing chromatograms of ms8-DelG/MS8 plant; (D,E) The sequencing chromatograms of ms8-InA/MS8 plant. In (B,D), PCR products were directly sequenced. In (C,E), PCR products were cloned into pEASY®-T1 Simple Cloning vectors (TransGen, Beijing, China) and the randomly selected individual clone was sequenced. The PAM motif is underlined and in bold. [file Image_1.JPEG]
