# Supplementary material for: Generation of Transgene-Free Maize Male Sterile Lines Using the CRISPR/Cas9 System
Source: Front Plant Sci. 2018 Sep 7;9:1180. doi: 10.3389/fpls.2018.01180 (PMC6137208; doi:10.3389/fpls.2018.01180)
Supplement: TABLE S1 — Primers used in this study. [file Table_1.docx]

**Supplementary Table 1 Primers used in this study**

| Primer name | Primer Sequence (5’-3’) | Function |
| --- | --- | --- |
| MS8ex2-crRNA-F | AGCAGCTGTCCGGGAAGGCCGTCG | The target site |
| MS8ex2-crRNA-R | AAACCGACGGCCTTCCCGGACAGC | The target site |
| Cas9-BsmBI-F | CTCGTCTCCCATGTCCGAGAGGAAGAGGA | Amplify the full-length Cas9 fragment |
| Cas9-BstEII-19R | GGGTGACCCTCAAGCGTAGTCTGGGACGTCGTATGGGTACCTCTTGCCGATGGCCTTC | Amplify the full-length Cas9 fragment |
| F1 | ATGATCAAGTTCAGGGGCCA | Amplify Cas9 fragment |
| R1 | CATCTTCTCCAGGATCGGCT | Amplify Cas9 and 35S::Cas9 fragment |
| F6 | TGATCTGCAGGAGGGTCG | Amplify zmU3::sgRNA fragment |
| R6 | CGACTCGGTGCCACTTTT | Amplify zmU3::sgRNA fragment |
| F5 | CCACTATCCTTCGCAAGACC | Amplify 35S::Bar and 35S::Cas9 fragment |
| R5 | GAAGTCCAGCTGCCAGAAAC | Amplify 35S::Bar fragment |
| F4 | TGATCCACCAGTCCATCACC | Amplify Cas9nos fragment |
| R4 | CGACAAGAAAACGCCAGGAA | Amplify Cas9nos fragment |
| F2 | AGCCGATCCTGGAGAAGATG | Amplify Cas9 fragment |
| R2 | GGAGTTCTTCTGGCCCTTCT | Amplify Cas9 fragment |
| F3 | GACGACTCCCTGACCTTCA | Amplify Cas9 fragment |
| R3 | GAGTTCCTCTTCGGCAGGAT | Amplify Cas9 fragment |
| MS8-crRNA-KZF | CGGCACATAAATCGTCTCCG | Amplify the target flanking fragment |
| MS8-crRNA-KZR | AACACGAATGCTCTTCTCGC | Amplify the target flanking fragment |
| MS8-ex2CXF | GGATTCCTGCTAGTGCTTCG | Sequence target site |
| MS8ex2offtargtJCJ | TTAGATGCGAGGCTGGATGT | Amplify and sequence the off-target flanking fragment |
| MS8ex2offtargtJCR | TGTGAGGGGCTGTACAAGAG | Amplify and sequence the off-target flanking fragment |
| Chr6offtargt-F | GTAGCCTCCAGTCATCGGAT | Amplify and sequence the off-target flanking fragment |
| Chr6offtargt-R | AGTCTTTCACTCAACCAGGTCT | Amplify and sequence the off-target flanking fragment |
| Chr4offtargt-2F | CATGAGTGGGGCGAGATAGT | Amplify and sequence the off-target flanking fragment |
| Chr4offtargt-2R | CCTCCTGCACCTCTTCTACC | Amplify and sequence the off-target flanking fragment |
| Chr8offtargt-2F | GCCTCACCACTTTTCAGACT | Amplify and sequence the off-target flanking fragment |
| Chr8offtargt-2R | ATGCTCAAATCGCCGTTCAG | Amplify and sequence the off-target flanking fragment |
