# Supplementary material for: Generation of Transgene-Free Maize Male Sterile Lines Using the CRISPR/Cas9 System
Source: Front Plant Sci. 2018 Sep 7;9:1180. doi: 10.3389/fpls.2018.01180 (PMC6137208; doi:10.3389/fpls.2018.01180)
Supplement: TABLE S2 — The genotypes of F1 plants crossing between transgenic line H17 with inbred line Zong31. [file Table_2.docx]

**Supplementary Table 2. The genotypes of F_1_ plants crossing between transgenic line H17 with inbred line Zong31**

| F_1_ plants No. | Line | Genotype |
| --- | --- | --- |
| 1 | *ms8*-DelG-1 | *ms8-DelG*/*MS8* |
| 2 | *ms8*-InA-2 | *ms8-InA*/*MS8* |
| 3 | *ms8*-DelG-3 | *ms8-DelG*/*MS8* |
| 4 | *ms8*-DelG-4 | *ms8-DelG*/*MS8* |
| 5 | *ms8*-DelG-5 | *ms8-DelG*/*MS8* |
| 6 | *ms8*-InA-6 | *ms8-InA*/*MS8* |
| 7 | *ms8*-DelG-7 | *ms8-DelG*/*MS8* |
| 8 | *ms8*-DelG-8 | *ms8-DelG*/*MS8* |
| 9 | *ms8*-InA-9 | *ms8-InA*/*MS8* |
| 10 | *ms8*-InA-10 | *ms8-InA*/*MS8* |
| 11 | *ms8*-DelG-11 | *ms8-DelG*/*MS8* |
| 12 | *ms8*-DelG-12 | *ms8-DelG*/*MS8* |

*DelG* indicates the new allele with a guanine nucleotide deletion, and *InA* indicates the new allele with a adenine nucleotide insertion.
