# Supplementary material for: Generation of Transgene-Free Maize Male Sterile Lines Using the CRISPR/Cas9 System
Source: Front Plant Sci. 2018 Sep 7;9:1180. doi: 10.3389/fpls.2018.01180 (PMC6137208; doi:10.3389/fpls.2018.01180)
Supplement: TABLE S3 — The genotypes of the off-target site with a single nucleotide difference. [file Table_3.docx]

**Supplementary Table 3. The genotypes of the off-target site with a single nucleotide difference**

| **Male sterile plant No.** | **genotype** |
| --- | --- |
| *ms8*-1 | *DelC/+* |
| *ms8*-2 | *DelC*/*+* |
| *ms8*-3 | *DelC*/*DelC* |
| *ms8*-4 | *DelC*/*DelC* |
| *ms8*-5 | *DelC*/*+* |
| *ms8*-6 | *DelC*/*+* |
| *ms8*-7 | *DelC*/*DelC* |
| *ms8*-8 | *DelC*/*DelC* |
| *ms8*-9 | *DelC*/*DelC* |
| *ms8*-10 | *DelC*/*+* |
| *ms8*-11 | *DelC*/*+* |
| *ms8*-12 | *DelC*/*DelC* |
| *ms8*-13 | *DelC*/*+* |
| *ms8*-14 | *+*/*+* |
| *ms8*-15 | *DelC*/*+* |
| *ms8*-16 | *DelC*/*DelC* |
| *ms8*-17 | *DelC*/*DelC* |
| *ms8*-18 | *+*/*+* |
| *ms8*-19 | *DelC*/ *+* |
| *ms8*-20 | *+*/*+* |
| *ms8*-21 | *DelC*/*+* |
| *ms8*-22 | *+*/*+* |
| *ms8*-23 | *+*/*+* |
| *ms8*-24 | No detection |

The *DelC* indicates the new allele with a cytosine nucleotide deletion.
