# Supplementary material for: Generation of Transgene-Free Maize Male Sterile Lines Using the CRISPR/Cas9 System
Source: Front Plant Sci. 2018 Sep 7;9:1180. doi: 10.3389/fpls.2018.01180 (PMC6137208; doi:10.3389/fpls.2018.01180)
Supplement: Supplementary file 5 [file Data_Sheet_1.DOCX]

1. **gRNA construct information**

*Hin*dIII

AAGCTTAGTGTACTTACTAGTAGTTTAGCCACCATTACCCAAATGCTTTCGAGCTTGTATTAAGACTTCCTAAGCTGAGCATCATCACTGATCTGCAGGAGGGTCGCTTCGCTGCCAAGATCAACAGCAACCATGTGGCGGCAACATCCAGCATTGCACATGGGCTAAAGATTGAGCTCTGTGCCAAGTGTGAGCTGCAACCATCTAGGGATCAGCTGAGTTTATCAGTCTTTCCTTTTTTTCATTCTGGTGAGGCATCAAGCTACTACTGCCTCGATCGGTTGGACTTGGACCTGAAGCCCACATGTAGGATACCAGAATGGACCGACCCAGGACGTAGTGCCACCTCGGTTGTCACACTGCGTAGAAGCCAGCTTAAAAATTTAGCTTTGGTGACTCACAGCACGAGACCAACAGGTCTCA**GTTTTAGA**

*Bsa*I *Bsa*I sgRNA

**GCTAGAAATAGCAAGTTAAAATAAGGCTAGTCCGTTATCAACTTGAAAAAGTGGCACCGAGTCGGTGCTT TTTTT**GGATCC

*Bam*HI

maize U3 promoter (399bp)

A transcriptional site

1. **Cas9 protein sequence**

MSERKRREKLDKKYSIGLDIGTNSVGWAVITDEYKVPSKKFKVLGNTDRHSIKKNLIGALLFDSGETAEATRLKRTARRRYTRRKNRICYLQEIFSNEMAKVDDSFFHRLEESFLVEEDKKHERHPIFGNIVDEVAYHEKYPTIYHLRKKLVDSTDKADLRLIYLALAHMIKFRGHFLIEGDLNPDNSDVDKLFIQLVQTYNQLFEENPINASGVDAKAILSARLSKSRRLENLIAQLPGEKKNGLFGNLIALSLGLTPNFKSNFDLAEDAKLQLSKDTYDDDLDNLLAQIGDQYADLFLAAKNLSDAILLSDILRVNTEITKAPLSASMIKRYDEHHQDLTLLKALVRQQLPEKYKEIFFDQSKNGYAGYIDGGASQEEFYKFIKPILEKMDGTEELLVKLNREDLLRKQRTFDNGSIPHQIHLGELHAILRRQEDFYPFLKDNREKIEKILTFRIPYYVGPLARGNSRFAWMTRKSEETITPWNFEEVVDKGASAQSFIERMTNFDKNLPNEKVLPKHSLLYEYFTVYNELTKVKYVTEGMRKPAFLSGEQKKAIVDLLFKTNRKVTVKQLKEDYFKKIECFDSVEISGVEDRFNASLGTYHDLLKIIKDKDFLDNEENEDILEDIVLTLTLFEDREMIEERLKTYAHLFDDKVMKQLKRRRYTGWGRLSRKLINGIRDKQSGKTILDFLKSDGFANRNFMQLIHDDSLTFKEDIQKAQVSGQGDSLHEHIANLAGSPAIKKGILQTVKVVDELVKVMGRHKPENIVIEMARENQTTQKGQKNSRERMKRIEEGIKELGSQILKEHPVENTQLQNEKLYLYYLQNGRDMYVDQELDINRLSDYDVDHIVPQSFLKDDSIDNKVLTRSDKNRGKSDNVPSEEVVKKMKNYWRQLLNAKLITQRKFDNLTKAERGGLSELDKAGFIKRQLVETRQITKHVAQILDSRMNTKYDENDKLIREVKVITLKSKLVSDFRKDFQFYKVREINNYHHAHDAYLNAVVGTALIKKYPKLESEFVYGDYKVYDVRKMIAKSEQEIGKATAKYFFYSNIMNFFKTEITLANGEIRKRPLIETNGETGEIVWDKGRDFATVRKVLSMPQVNIVKKTEVQTGGFSKESILPKRNSDKLIARKKDWDPKKYGGFDSPTVAYSVLVVAKVEKGKSKKLKSVKELLGITIMERSSFEKNPIDFLEAKGYKEVKKDLIIKLPKYSLFELENGRKRMLASAGELQKGNELALPSKYVNFLYLASHYEKLKGSPEDNEQKQLFVEQHKHYLDEIIEQISEFSKRVILADANLDKVLSAYNKHRDKPIREQAENIIHLFTLTNLGAPAAFKYFDTTIDRKRYTSTKEVLDATLIHQSITGLYETRIDLSQLGGDMISESLRKAIGKR-

Nuclear location signal

N terminal：MSERKRREKL

C terminal：MISESLRKAIGKR

1. **Codon optimized Cas9 DNA sequence**

*Bsm*BI NLS

CGTCTCCC**ATG**TCCGAGAGGAAGAGGAGGGAGAAGCTGGACAAGAAGTACTCCATCGGCCTGGACATCGGCACCA

ACTCCGTGGGCTGGGCCGTGATCACCGACGAGTACAAGGTGCCGTCCAAGAAGTTCAAGGTGCTGGGCAACACCGACAGGCACTCCATCAAGAAGAACCTGATCGGCGCCCTGCTGTTCGACTCCGGCGAGACCGCCGAGGCCACCAGGCTGAAGAGGACCGCCAGGAGGAGGTACACCAGGAGGAAGAACAGGATCTGCTACCTGCAGGAGATCTTCTCCAACGAGATGGCCAAGGTGGACGACTCCTTCTTCCACAGGCTGGAGGAGTCCTTCCTGGTGGAGGAGGACAAGAAGCACGAGAGGCACCCGATCTTCGGCAACATCGTGGACGAGGTGGCCTACCACGAGAAGTACCCGACCATCTACCACCTGAGGAAGAAGCTGGTGGACTCCACCGACAAGGCCGACCTGAGGCTGATCTACCTGGCCCTGGCCCACATGATCAAGTTCAGGGGCCACTTCCTGATCGAGGGCGACCTGAACCCGGACAACTCCGACGTGGACAAGCTGTTCATCCAGCTGGTGCAGACCTACAACCAGCTGTTCGAGGAGAACCCGATCAACGCCTCCGGCGTGGACGCCAAGGCCATCCTGTCCGCCAGGCTGTCCAAGTCCAGGAGGCTGGAGAACCTGATCGCCCAGCTGCCGGGCGAGAAGAAGAACGGCCTGTTCGGCAACCTGATCGCCCTGTCCCTGGGCCTGACCCCGAACTTCAAGTCCAACTTCGACCTGGCCGAGGACGCCAAGCTGCAGCTGTCCAAGGACACCTACGACGACGACCTGGACAACCTGCTGGCCCAGATCGGCGACCAGTACGCCGACCTGTTCCTGGCCGCCAAGAACCTGTCCGACGCCATCCTGCTGTCCGACATCCTGAGGGTGAACACCGAGATCACCAAGGCCCCGCTGTCCGCCTCCATGATCAAGAGGTACGACGAGCACCACCAGGACCTGACCCTGCTGAAGGCCCTGGTGAGGCAGCAGCTGCCGGAGAAGTACAAGGAGATCTTCTTCGACCAGTCCAAGAACGGCTACGCCGGCTACATCGACGGCGGCGCCTCCCAGGAGGAGTTCTACAAGTTCATCAAGCCGATCCTGGAGAAGATGGACGGCACCGAGGAGCTGCTGGTGAAGCTGAACAGGGAGGACCTGCTGAGGAAGCAGAGGACCTTCGACAACGGCTCCATCCCGCACCAGATCCACCTGGGCGAGCTGCACGCCATCCTGAGGAGGCAGGAGGACTTCTACCCGTTCCTGAAGGACAACAGGGAGAAGATCGAGAAGATCCTGACCTTCCGCATCCCGTACTACGTGGGCCCGCTGGCCAGGGGCAACTCCAGGTTCGCCTGGATGACCAGGAAGTCCGAGGAGACCATCACCCCGTGGAACTTCGAGGAGGTGGTGGACAAGGGCGCCTCCGCCCAGTCCTTCATCGAGAGGATGACCAACTTCGACAAGAACCTGCCGAACGAGAAGGTGCTGCCGAAGCACTCCCTGCTGTACGAGTACTTCACCGTGTACAACGAGCTGACCAAGGTGAAGTACGTGACCGAGGGCATGAGGAAGCCGGCCTTCCTGTCCGGCGAGCAGAAGAAGGCCATCGTGGACCTGCTGTTCAAGACCAACAGGAAGGTGACTGTGAAGCAGCTGAAGGAGGACTACTTCAAGAAGATCGAGTGCTTCGACTCCGTGGAGATCTCCGGCGTGGAGGACAGGTTCAACGCCTCCCTGGGCACCTACCACGACCTGCTGAAGATCATCAAGGACAAGGACTTCCTGGACAACGAGGAGAACGAGGACATCCTGGAGGACATCGTGCTGACCCTGACCCTGTTCGAGGACAGGGAGATGATCGAGGAGAGGCTGAAGACCTACGCCCACCTGTTCGACGACAAGGTGATGAAGCAGCTGAAGAGGAGGAGGTACACCGGCTGGGGCAGGCTGTCCAGGAAGCTGATCAACGGCATCAGGGACAAGCAGTCCGGCAAGACCATCCTGGACTTCCTGAAGTCCGACGGCTTCGCCAACAGGAACTTCATGCAGCTGATCCACGACGACTCCCTGACCTTCAAGGAGGACATCCAGAAGGCCCAGGTGTCCGGCCAGGGCGACTCCCTGCACGAGCACATCGCCAACCTGGCCGGCTCCCCGGCCATCAAGAAGGGCATCCTGCAGACCGTGAAGGTGGTGGACGAGCTGGTGAAGGTGATGGGCAGGCACAAGCCGGAGAACATCGTGATCGAGATGGCCAGGGAGAACCAGACCACCCAGAAGGGCCAGAAGAACTCCAGGGAGAGGATGAAGAGGATCGAGGAGGGCATCAAGGAGCTGGGCTCCCAGATCCTGAAGGAGCACCCGGTGGAGAACACCCAGCTGCAGAACGAGAAGCTGTACCTGTACTACCTGCAGAACGGCAGGGACATGTACGTGGACCAGGAGCTGGACATCAACAGGCTGTCCGACTACGACGTGGACCACATCGTGCCGCAGTCCTTCCTGAAGGACGACTCCATCGACAACAAGGTGCTGACCAGGTCCGACAAGAACAGGGGCAAGTCCGACAACGTGCCGTCCGAGGAGGTGGTGAAGAAGATGAAGAACTACTGGAGGCAGCTGCTGAACGCCAAGCTGATCACCCAGAGGAAGTTCGACAACCTGACCAAGGCCGAGAGGGGCGGCCTGTCCGAGCTGGACAAGGCCGGCTTCATCAAGAGGCAGCTGGTGGAGACCAGGCAGATCACCAAGCACGTGGCCCAGATCCTGGACTCCAGGATGAACACCAAGTACGACGAGAACGACAAGCTGATCAGGGAGGTGAAGGTGATCACCCTGAAGTCCAAGCTGGTGTCCGACTTCAGGAAGGACTTCCAGTTCTACAAGGTGAGGGAGATCAACAACTACCACCACGCCCACGACGCCTACCTGAACGCCGTGGTGGGCACCGCCCTGATCAAGAAGTACCCGAAGCTGGAGTCCGAGTTCGTGTACGGCGACTACAAGGTGTACGACGTGAGGAAGATGATCGCCAAGTCCGAGCAGGAGATCGGCAAGGCCACCGCCAAGTACTTCTTCTACTCCAACATCATGAACTTCTTCAAGACCGAGATCACCCTGGCCAACGGCGAGATCAGGAAGAGGCCGCTGATCGAGACCAACGGCGAGACCGGCGAGATCGTGTGGGACAAGGGCAGGGACTTCGCCACCGTGAGGAAGGTGCTGTCCATGCCGCAGGTGAACATCGTGAAGAAGACCGAGGTGCAGACCGGCGGCTTCTCCAAGGAGTCCATCCTGCCGAAGAGGAACTCCGACAAGCTGATCGCCAGGAAGAAGGACTGGGACCCGAAGAAGTACGGCGGCTTCGACTCCCCGACCGTGGCCTACTCCGTGCTGGTGGTGGCCAAGGTGGAGAAGGGCAAGTCCAAGAAGCTGAAGTCCGTGAAGGAGCTGCTGGGCATCACCATCATGGAGAGGTCCTCCTTCGAGAAGAACCCGATCGACTTCCTGGAGGCCAAGGGCTACAAGGAGGTGAAGAAGGACCTGATCATCAAGCTGCCGAAGTACTCCCTGTTCGAGCTGGAGAACGGCAGGAAGAGGATGCTGGCCTCCGCCGGCGAGCTGCAGAAGGGCAACGAGCTGGCCCTGCCGTCCAAGTACGTGAACTTCCTGTACCTGGCCTCCCACTACGAGAAGCTGAAGGGCTCCCCGGAGGACAACGAGCAGAAGCAGCTGTTCGTGGAGCAGCACAAGCACTACCTGGACGAGATCATCGAGCAGATCTCCGAGTTCTCCAAGAGGGTGATCCTGGCCGACGCCAACCTGGACAAGGTGCTGTCCGCCTACAACAAGCACAGGGACAAGCCGATCAGGGAGCAGGCCGAGAACATCATCCACCTGTTCACCCTGACCAACCTGGGCGCCCCGGCCGCCTTCAAGTACTTCGACACCACCATCGACAGGAAGAGGTACACCTCCACCAAGGAGGTGCTGGACGCCACCCTGATCCACCAGTCCATCACCGGCCTGTACGAGACCAGGATCGACCTGTCCCAGCTGGGCGGCGACATGATCTCCGAGTCCCTGAGGAAGGCCATCGGCAAGAGGTGAGGTGACC NLS

*Bst*EII
